# Acute Gastric Ischemia in a Case of Small Bowel Obstruction

**DOI:** 10.7759/cureus.55113

**Published:** 2024-02-27

**Authors:** Nasser T Almarri, Rafaat J Elsaadawy, Ahmed M Alhumaidan

**Affiliations:** 1 General Surgery, Buraidah Central Hospital, Buraidah, SAU

**Keywords:** small bowel obstruction, outflow obstruction, gastric necrosis, acute gastric dilatation, acute gastric ischemia

## Abstract

Gastric ischemia is a relatively rare condition that can lead to severe or life-threatening outcomes. It can be caused by various etiological factors, including vascular occlusion, atherosclerosis, vasculitis, hypovolemic shock, cardiac failure, mesenteric ischemia, splanchnic vasoconstriction, and abdominal compartment syndrome. Furthermore, gastric dilation can be caused by volvulus and acute necrotizing gastritis. This condition may go unnoticed in the setting of intestinal obstruction. In this case report, we describe a 43-year-old female who presented with signs, symptoms, and radiological findings indicative of small bowel obstruction accompanied by a severely dilated stomach. Our aim is to highlight the importance of considering gastric ischemia in patients with small bowel obstruction and to demonstrate the outcomes of a surgical approach in such presentations.

## Introduction

Acute gastric ischemia resulting from intestinal obstruction is a rare yet complex condition. Vascular occlusion can arise from thrombotic events and inflammatory processes, leading to ischemia or total vascular occlusion [1]. Since nutrient transfer occurs in the intestine, the gastrointestinal system has abundant vascularization. Consequently, the diagnosis of acute gastric ischemia often goes unnoticed. Pathologic examination of gastric ischemia reveals contributing factors such as capillary dilatation and vascular congestion. Intestinal volvulus can also induce gastric ischemia, wherein bowel loops intertwine or twist, causing marked gastric distention that can also precipitate gastric ischemia, which may manifest with symptoms such as abdominal pain, distention, nausea, and vomiting, potentially progressing to necrosis [2]. Vascular insufficiency stands out as a major cause of gastric ischemia. This condition can be challenging to detect due to the stomach's abundant blood supply [3]. Within vascular insufficiency, venous occlusion is particularly perilous compared to arterial occlusion, as some studies indicate that if intragastric pressure exceeds > 20 cm H2O, it can impair the venous return of gastric veins [4,5]. It is crucial to consider gastric ischemia in cases of gastric distension, especially when associated with bowel obstruction, to prevent serious adverse outcomes like perforation of the stomach wall [6]. In this presentation, we discuss a case of gastric ischemia in the context of small bowel obstruction.

## Case presentation

**Figure 1:**
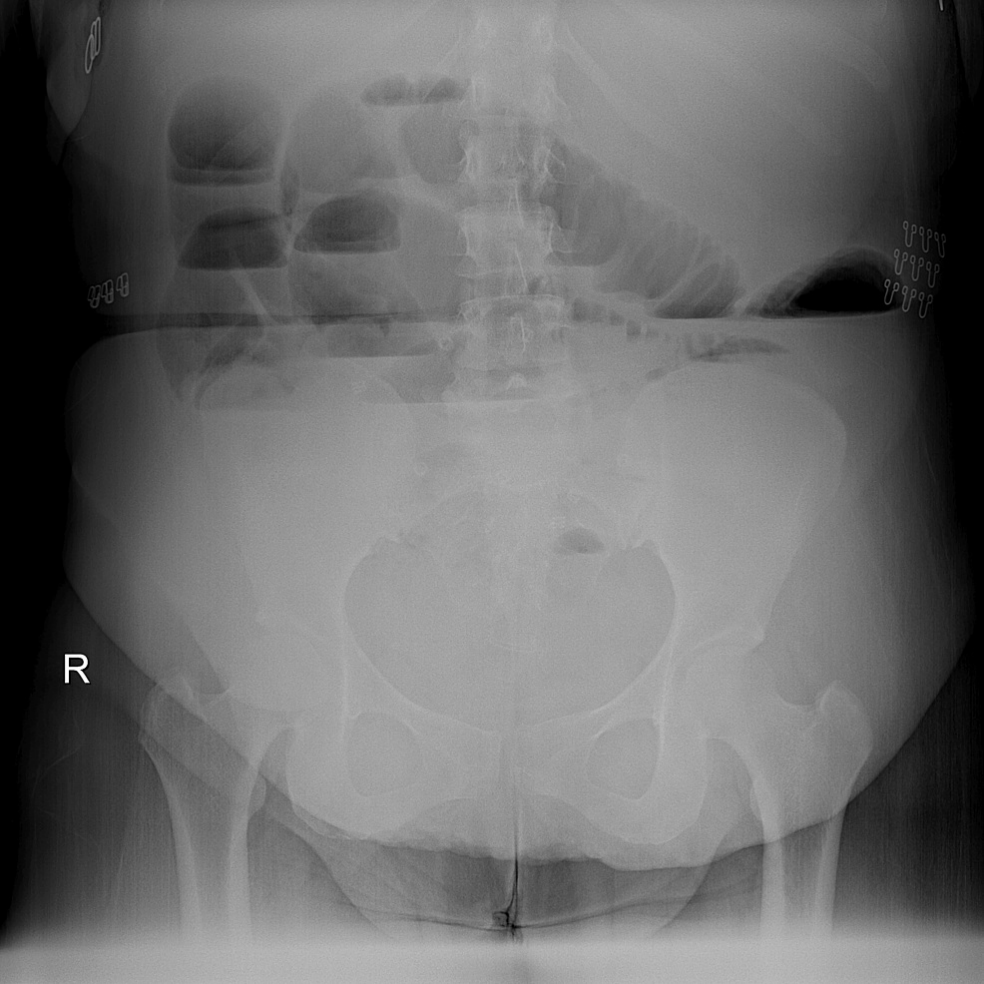
Erect abdominal X-ray shows air-fluid levels

A 43-year-old female was admitted to the emergency department with a history spanning over two days of abdominal discomfort, constipation, nausea, vomiting, and abdominal distention. The pain was localized to the upper abdominal region and was of moderate severity. The patient's medical records showed a diagnosis of hypothyroidism. Surgically, the patient had a history of an open appendectomy, repair of paraumbilical hernia with mesh, and four Cesarean sections. Upon clinical assessment, an abdominal distension with generalized tenderness markedly over the epigastrium, without evidence of abdominal rigidity, was found. The patient exhibited tachypnea and tachycardia and maintained normal blood pressure and mean arterial pressure (MAP). An erect abdominal X-ray revealed multiple air-fluid levels and a lack of air in the rectum (Figure 1). Laboratory results indicated an elevated leukocyte count, elevated serum amylase, and decreased serum sodium; venous blood gas sampling demonstrated a low pH, a high lactic acid level, and a decreased bicarbonate level with a low base deficit (Table 1). However, all coagulative profiles were within normal limits.

**Table 1:** Patient's laboratory results

Laboratory test	Result	Reference range
WBCs	14 x 10^3^/μL	4.5 - 11 x 10^3^/μL
Serum amylase	147.80 U/L	28-100 U/L
Serum sodium	115 mmol/L	136-146 mmol/L
pH	7.29	7.35-7.45
Lactic acid	6.3 mmol/L	0.5–1.6 mmol/L
Bicarbonate	13.1 mmol/L	22-26 mmol/L
Base deficit	-16.3 mmol/L	2.0 to -2.0 mmol/L

**Figure 2:**
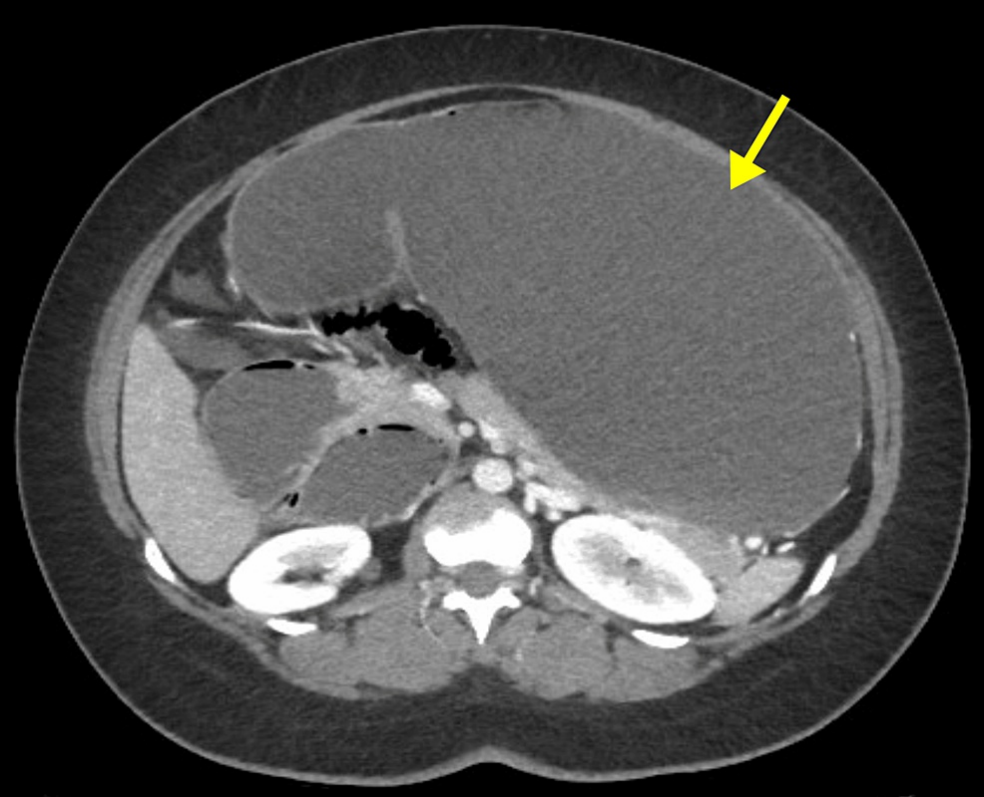
Abdominal CT with contrast shows a markedly distended stomach

**Figure 3:**
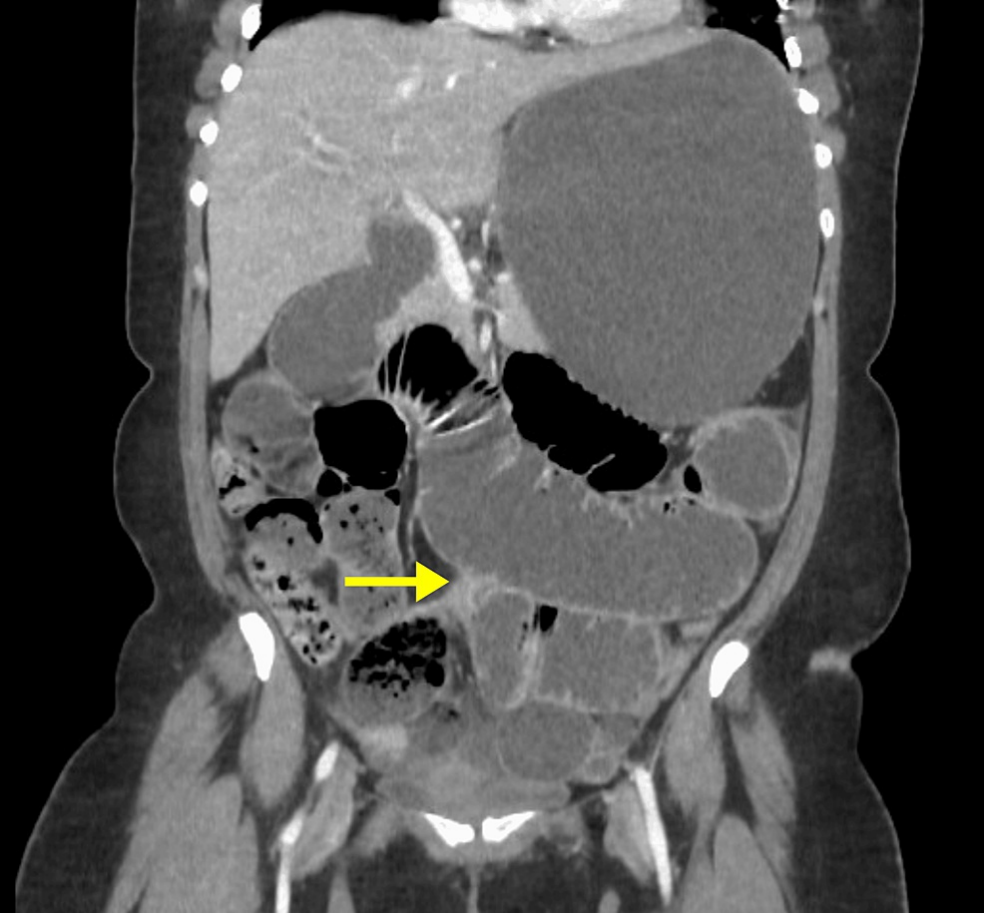
Abdominal CT with contrast shows the small bowel transition zone

**Figure 4:**
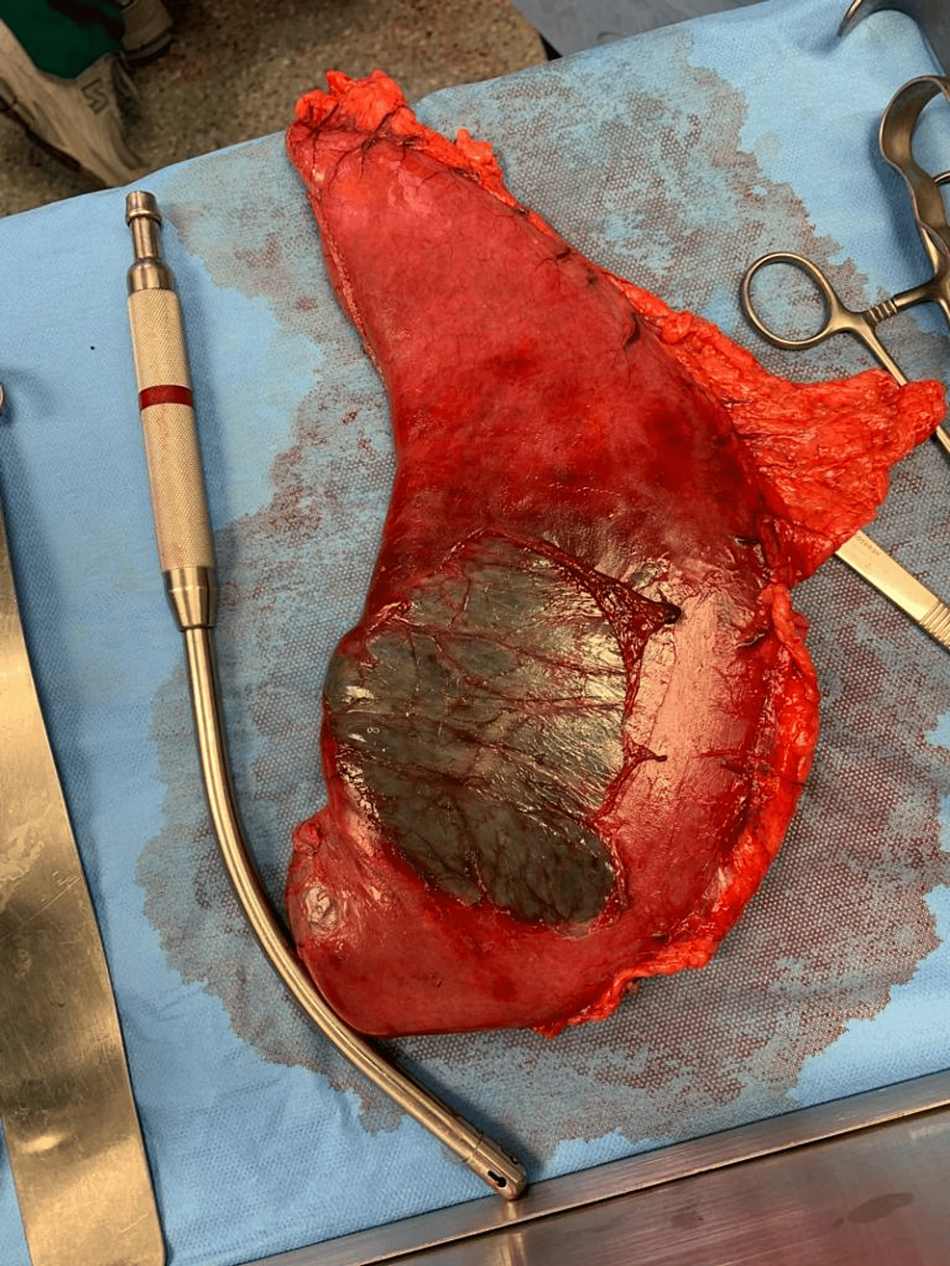
Resected part of the stomach with gangrene patch over the anterior aspect of the greater curvature

**Figure 5:**
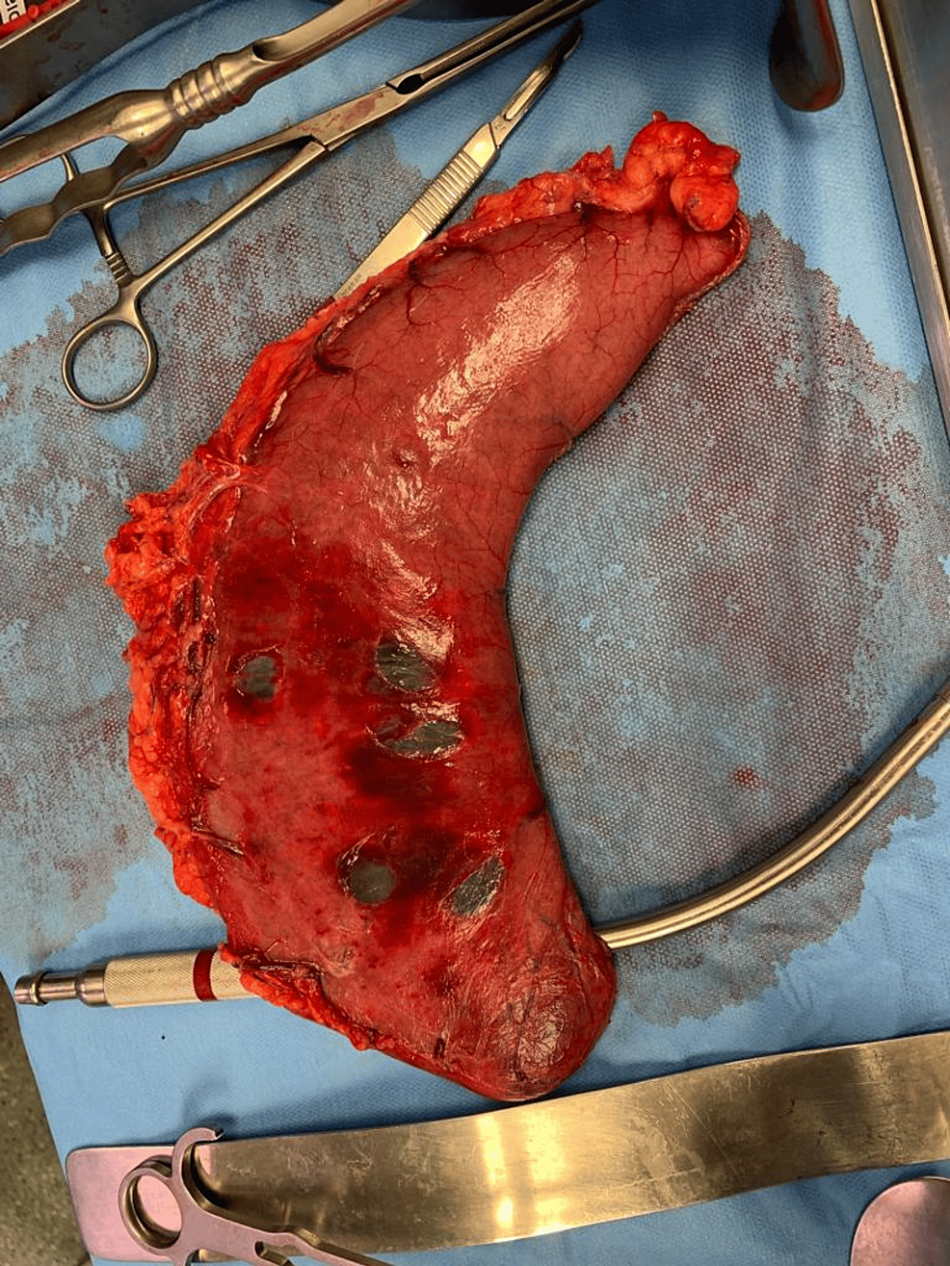
Resected part of the stomach with gangrene patches over the posterior aspect of the greater curvature

**Figure 6:**
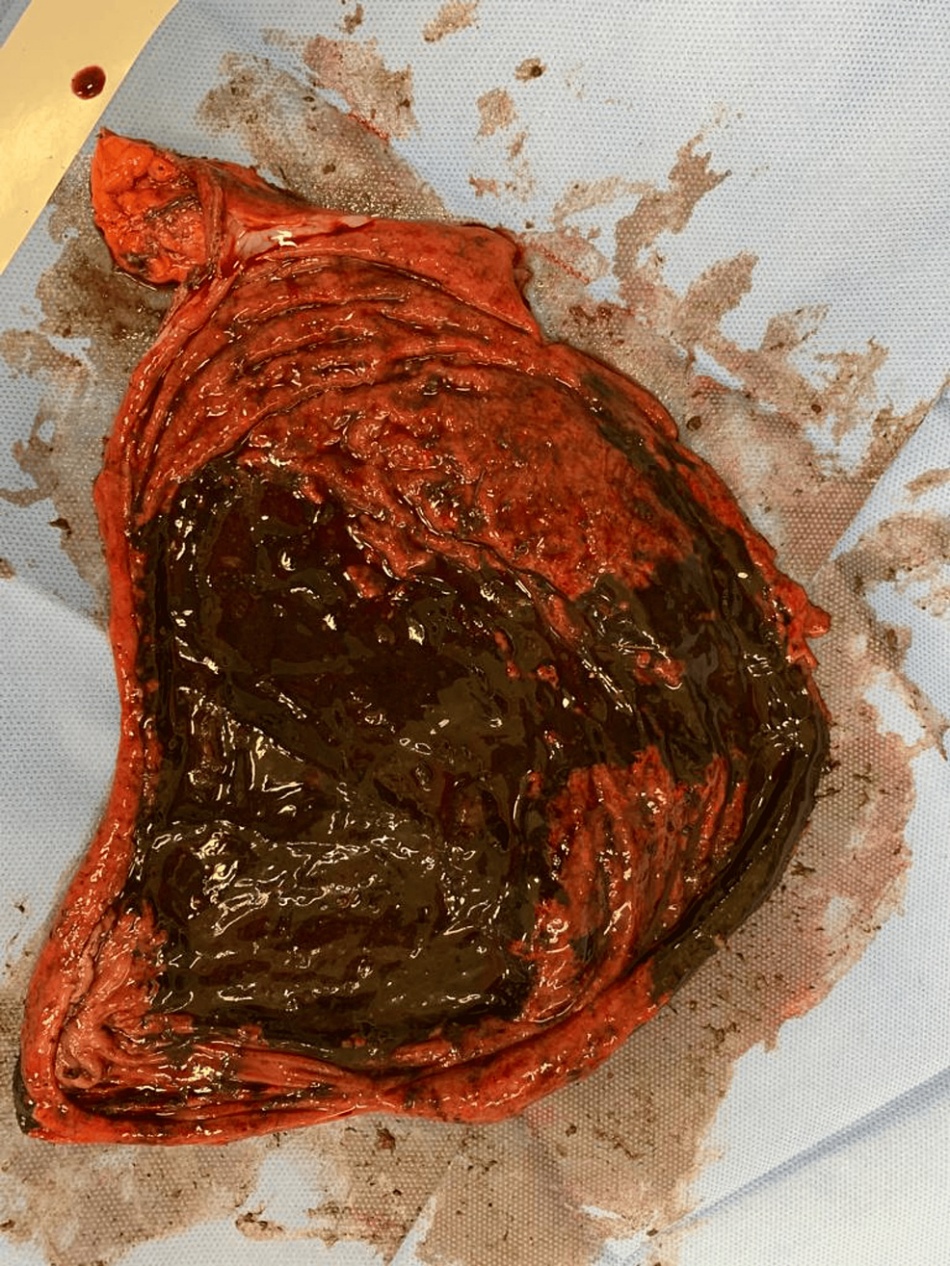
Gross picture of the incised wall of the stomach

CT scan revealed marked distension of the stomach (Figure 2) and small bowel (jejunum, proximal, and mid ileum) with sudden caliber reduction of the ileal lumen near the right iliac fossa (Ileocecal transition zone) (Figure 3). The maximum diameter of the distended bowel measured approximately 4.8 cm, while the large bowel was collapsed. A nasogastric tube (NGT) was inserted in the emergency department, which yielded 1400 ml of gastric content resembling coffee grounds. The patient was admitted and underwent emergent midline exploratory laparotomy. The small bowel exhibited distension without ischemic changes, and adhesiolysis of multiple bands reaching the transition zone around one meter from the ileocecal valve was performed. During the operation, stomach decompression yielded another 1200 ml of coffee ground fluid. A full exploration revealed a gangrenous patch over the anterior aspect of the greater curvature of the stomach (Figure 4), and the opening of the lesser sac showed patches of gangrene over the posterior aspect (Figure 5). A partial gastrectomy was performed with secondary interrupted sutures over the staple line, the short gastric artery was ligated, and the specimen was sent for histopathology (Figure 6). Two drains were inserted in the pelvic and left hypochondriac regions; then, mass abdomen closure was done.

Postoperatively, the patient was transferred intubated to the ICU. In the ICU, the patient exhibited instability in hemodynamic parameters and remained intubated for 12 days on inotropes. Feeding through NGT commenced gradually on day four postoperatively. The patient was extubated on day 12 and transferred to the ward on day 14 with NGT. An abdominal CT scan with oral and IV contrast was conducted for follow-up, revealing a non-distended stomach with no leakage. The NGT was removed, and a standard dietary regimen was initiated. On day 38, post-operation, the patient was discharged and visited the outpatient clinic after one week for follow-up without any complaints. A histopathological examination revealed pronounced vascular congestion and localized tissue infarction, with tissue changes consistent with gangrene and viable tissue margins.

## Discussion

Gastric ischemia arises from an insufficient supply of oxygen to the cells lining the gastrointestinal tract [7]. This condition can present in acute and chronic forms; however, as illustrated in this case study, the patient exhibited symptoms indicative of acute gastric ischemia. Intestinal obstructions can also precipitate gastric ischemia [8]. The stomach's extensive vascular network typically protects it from acute necrotic damage, making such an injury a rare occurrence. Acute gastric dilation is identified as one of the causes of gastric ischemia, with various factors contributing to its occurrence [9]. The existing literature indicates that trauma, electrolyte disturbances, eating disorders, gastric volvulus, small bowel obstruction, and diabetes mellitus are among the reasons behind gastric ischemia [10]. In this case study, the patient exhibited signs of bowel obstruction, as confirmed through physical examination, laboratory findings, and radiological imaging, including a CT scan. Although the CT scan revealed a markedly dilated stomach, the primary concern was the small bowel obstruction. This observation aligns with the existing literature, which suggests that such clinical presentations can be indicative of underlying gastric ischemia [11,12].

It is reported that the occlusion of principal gastric arteries alone does not induce gastric ischemia. In the setting of intestinal obstruction, the arterial supply to the bowel remains uninterrupted, leading to an increase in back pressure. This elevated pressure contributes to the development of bowel wall edema and potential intramural hemorrhage, which may manifest as coffee ground-like material in the nasogastric tube. The combination of increased back pressure and thickening of the bowel wall can compromise tissue perfusion, resulting in a cascade of events that includes intestinal ischemia, infarction, and ultimately necrosis [6]. Intragastric pressure exceeding 20 cm H2O impairs the venous return of gastric veins [6]. Notably, 3 liters of fluid can elevate stomach pressure to the point of venous outflow obstruction, as suggested by multiple case reports [13-15]. In our case, 1400 ml of coffee ground fluid was drained by NGT in the emergency room and another 1200 ml in the operating room. Gastric ischemia stemming from various causes can lead to critical and life-threatening outcomes such as rupture and hemorrhage, resulting in peritonitis, sepsis, and even death [16]. Studies on gastric volume in cadavers positioned in a sitting posture have shown that the administration of 4 liters of fluid can regularly cause tears in the mucous membranes along the lesser curvature [6]. Furthermore, according to Lee et al., the gastric capacity is 4 liters before perforation occurs [17]. Some patients displayed necrosis affecting the greater curvature or fundus, while the lesser curvature was preserved [18]. In our case, ischemic patches were observed on both the anterior and posterior aspects of the greater curvature.

Diagnosing acute gastric ischemia involves recognizing the clinical manifestations in patients, followed by radiological testing such as CT scans [19]. Prompt diagnostic assessment is crucial in preventing the progression of the condition; although it is rare, if undetected, it may lead to mortality [20]. Therapeutic approaches for acute gastric ischemia vary from medical management to surgery [21]. In the presented case, surgery, specifically partial gastrectomy, was considered the most suitable approach; however, this process requires adequate vascular perfusion and a significant time investment [22]. Partial resection is a surgical strategy for treating ischemia when the pathology involves patchy necrosis and gangrene, minimizing the chances of morbidity. It is recommended to consider limited resection as the initial therapeutic intervention for gastric ischemia if the conservative treatment is not a valid option [23].

## Conclusions

Acute ischemia of the stomach denotes the interruption of the blood supply to the organ. A pathologic examination of gastric ischemia reveals a few factors influencing the medical condition, including capillary dilation, vascular congestion, and, in some cases, necrosis. Vascular occlusion stands out as a major factor in gastric ischemia. Among instances of vascular insufficiency, venous occlusion poses greater complexity compared to arterial insufficiency. Excessive gastric dilation can also lead to gastric ischemia, characterized by rapid stomach enlargement. The diagnosis of acute gastric ischemia involves observing patients' clinical manifestations, such as vomiting, gastrointestinal tract bleeding, and nausea. Various radiological tests, including CT scans and ultrasounds, are considered standard for diagnostic assessment. Treatment strategies for acute gastric ischemia span from medical to surgical intervention, depending on the presentation. The diagnosis of gastric ischemia demands a rapid initiation of the management; although it is rare, it can be fatal. Gastric ischemia should be suspected in a patient with symptoms of bowel obstruction combined with an overly dilated stomach. Decompression with NGT, radiological tests, and surgical exploration are mandated to ensure gastric ischemia is not missed.
